# The biomechanics of fish skin: assessing puncture resistance to the dynamic predatory mechanism of cone snails

**DOI:** 10.1242/jeb.250634

**Published:** 2026-01-02

**Authors:** Bishal Baskota, Bingyang Zhang, Philip S. L. Anderson

**Affiliations:** ^1^ Department of Evolution, Ecology, and Behavior, School of Integrative Biology, University of Illinois Urbana-Champaign, 505 S. Goodwin Ave., Urbana, IL 61801, USA; ^2^ Ingram School of Engineering, Texas State University, San Marcos, TX 78666, USA

**Keywords:** Fish integument, Puncture tools, Damage reduction, Biological puncture, Unconstrained target

## Abstract

In aquatic species such as fish, the integumentary system, comprising skin and scales, serves as a crucial defense against puncture from high-velocity impacts. While previous studies have focused on quasistatic puncture behavior and constrained targets, here we investigated the less-studied dynamic puncture behavior in both constrained and unconstrained fish integument samples. We used cone snails as a model organism, which utilize a ballistic radular tooth to penetrate and paralyze prey. Our dynamic puncture experiments demonstrate that fish integument effectively mitigates damage from predatory mechanisms at biologically relevant speeds. While higher velocities typically result in deeper penetration, puncture performance is significantly reduced at lower speeds in unconstrained targets. These findings reveal the protective function and biomechanical efficiency of fish integument, with high puncture resistance attributed to material properties, momentum transfer and mobility. Our results highlight the adaptive strategies of cone snails in overcoming these defenses with greater velocity and energy.

## Teaser

**Summary:** High-speed puncture experiments illustrate how thin fish skin can reduce damage during predation, while unconstrained fish targets require higher impact speed for full penetration.

## INTRODUCTION

In many organisms, the integumentary system serves as the outermost protective layer against environmental pressures and mechanical hazards posed by predators utilizing puncture tools (Anderson et al., 2016; Anderson, 2018; Zhang et al., 2024). Most previous research on biological puncture focused on controlled quasi-static conditions (Zhu et al., 2012, 2013; Wang et al., 2016; Minicozzi et al., 2019; Ghods et al., 2019; Yaseen et al., 2021; Scali et al., 2019; Galloway and Porter, 2021), yet punctures in nature often occur at dynamic speeds (Zhang and Anderson, 2022, 2023; Anderson et al., 2016; Anderson, 2018; Anderson and Kawano, 2022). Research by Zhang et al. (2024) on layered porcine skin-adipose tissues has shown that material properties of the outer skin layer, despite its thinness compared with the subcutaneous tissue, play a critical role in providing puncture resistance and reducing internal tissue damage under dynamic conditions (Zhang et al., 2024). In contrast, aquatic species such as fish possess a unique integument structure, often with stiff and tough scales covering the skin layer. Over time, fish integument has evolved into a natural defense in aquatic environments against high-speed predatory strikes from sharp teeth, spines, and other piercing tools (Elliott, 2011; Sherman et al., 2017). The skin and scales combine properties such as elasticity and toughness to distribute impact forces and mitigate damage during predatory encounters (Zhang et al., 1994; Finlay, 1970; Lanir and Fung, 1974; Wainwright et al., 1978; Grear et al., 2018). However, the effect of kinematics on the damage resistance of the fish integument remains largely unexplored. In this study, we addressed this gap by quantifying the damage reduction capability of the fish integument during biologically relevant high-speed puncture.

While most studies on dynamic impact in biology have focused on testing with constrained targets – samples that are rigidly fixed or have constrained movement along the direction of impact (Zhu et al., 2012, 2013; Wang et al., 2016; Minicozzi et al., 2019; Ghods et al., 2019; Yaseen et al., 2021; Scali et al., 2019; Galloway and Porter, 2021) – high-speed puncture systems in nature (≳10 m s^−1^) (Zhang and Anderson, 2023) often attack unconstrained targets that are free to move upon impact. This will introduce unique variables such as momentum transfer and target mobility. For example, in aquatic environments, fish are free to locomote or alter their body position to evade predatory attacks. Even when struck, their unconstrained bodies can still be displaced passively. As such, the impact momentum will be partially redirected and transferred to the target's motion, therefore dissipating the energy available for penetration and potentially reducing the depth of puncture and the effectiveness of the attack (Anderson et al., 2019).

In this study, we present a biologically relevant analysis of dynamic puncture behavior on fish skin, using cone snails as a model species. Cone snails are opportunistic hunters known for their high-speed puncture mechanism (Olivera et al., 2015; Kapil et al., 2025; Salisbury et al., 2010), capable of delivering venom through a harpoon-like radular tooth (Schulz et al., 2004) and striking at an average peak velocity of 19.3 m s^−1^ (Schulz et al., 2019). This kinematic measurement, combined with the shape of the cone snail radular teeth, provides a biologically relevant framework for investigating the dynamic puncture behavior of fish integument. Using controlled puncture experiments, we examined the effects of fish skin layers and puncture rates on puncture resistance. We explored the interplay between applied puncture kinematics and momentum transfer on unconstrained fish targets during simulated dynamic biological puncture. We hypothesized that a sufficiently high attack speed, as seen in cone snail hunting strategy, is necessary to achieve successful puncture without simply deforming or displacing the target.

## MATERIALS AND METHODS

### Sample preparation

**Fig. 1.**
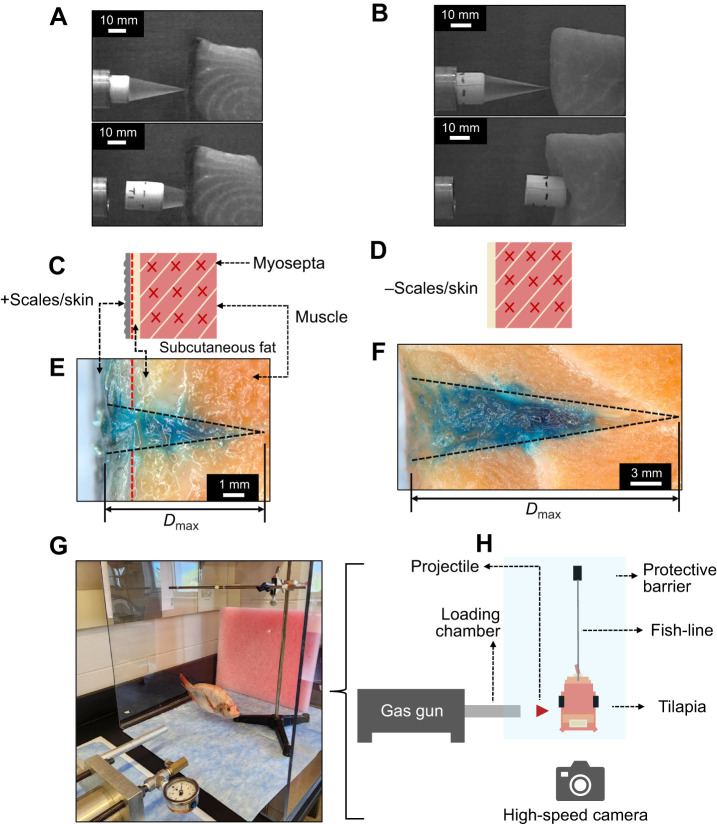
**Representative dynamic puncture tests and their damage morphologies.** (A,C,E) Salmon sample with (+) scales and skin; (B,D,F) salmon sample without (−) scales and skin. (A,B) High-speed still-frame images of dynamic puncture tests, showing the onset of impact (top) and the point of maximum penetration (bottom), respectively. Scale bars: 10 mm. (C,D) Schematic diagram of the salmon samples and their structures. (E,F) Microscopic images of undeformed fractured surfaces. The dashed red lines indicate the interface between the outer-skin and subcutaneous-tissue layers. *D*_max_, depth of puncture. (G) Dynamic puncture tests on unconstrained targets. (H) Schematic diagram of the experimental setup (front view). The tilapia is suspended and positioned perpendicularly to the puncture direction.

**Fig. 2.**
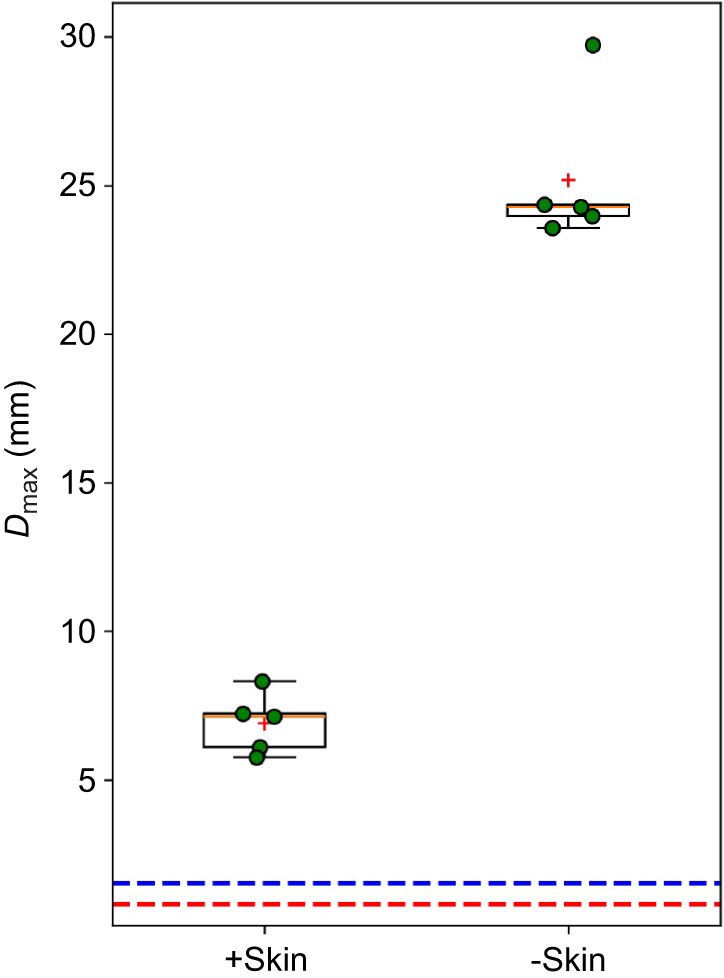
**Dynamic puncture results for layered salmon tissues, obtained at an average velocity of 18.5±1.1 m s^−1^**. For the box plots, the top and bottom edges of each box correspond to the 75th and 25th percentiles; the center yellow line indicates the median and the red cross indicates the mean; the whiskers indicate the scatter of the dataset. Data points of five individual tests are illustrated by green circles for each condition. The horizontal dashed blue line at a depth of 1.51 mm denotes the threshold for a successful puncture. The horizontal dashed red line at a depth of 0.795 mm represents the minimum penetration to reach the subcutaneous layer. A two-sample *t*-test confirmed significant differences in puncture depths between conditions (*P*<0.0001).

**Fig. 3.**
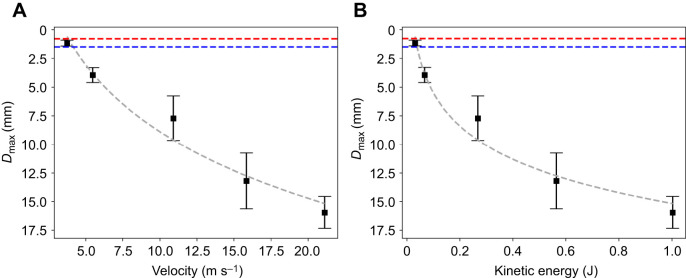
**Puncture performance in unconstrained tilapia samples.** Data were obtained at different puncture rates. (A) Velocity as a function of *D*_max_. (B) Kinetic energy as a function of *D*_max_. Data points and error bars represent the mean and standard deviation obtained from at least two individual tests. Controlled puncture test conditions: cusp angle θ≈23.0 deg; tip radius *r*≈92*.*6 µm. The fitted curve uses a logarithmic regression to indicate the trend. The horizontal dashed blue and red lines represent the thresholds for a successful puncture, as detailed in Fig. 2.

Salmon and tilapia samples were obtained from Costco (Champaign, IL, USA). Salmon samples were standard boneless fillets taken from the central body region along the lateral side of the fish between the head and tail. The fish samples were frozen within 1 h and kept frozen for ∼24 h. Prior to sample preparation, the fish samples were thawed for ∼2 h to ensure adequate softening of tissues. The salmon samples were then cut into uniform cubic sections with muscle fibers oriented orthogonally to the direction of puncture (Fig. 1) and movement constrained in the same direction. For the skinless testing condition, the skin was removed by cutting around its edges and gently pulling it off the meat. All testing was conducted within a 1 h window after the samples had been thawed and prepared. Tilapia samples were unconstrained and tested directly without any processing after thawing (Fig. 1G). Because of time constraints, a small subset of the samples was subjected to a second freeze–thaw cycle before testing. However, we did not observe any significant impact on the corresponding test results (Figs 2 and 3).

### Dynamic puncture method

Dynamic puncture tests were performed following documented protocols (Zhang and Anderson, 2022, 2023) using a compressed air cannon [Ballistic Loading and Structural Testing Lab (BLAST), NC State University, USA]. The 3D-printed projectiles used for puncture [Form 3 stereolithography (SLA) 3D printer, Formlabs Inc., clear resin, FLGPCL04], featured a conical shaped body with a cusp angle, θ*=*23*.*0 deg, and an average tip radius *r*≈92*.*6 µm after surface polishing (2000 grit sandpaper). The cusp angle measurement was extracted from scanning electron microscope (SEM) image analysis of cone snail radular harpoons documented in previous research (Kohn et al., 1999; Franklin et al., 2007) using ImageJ (Fig. S1). Specifically, *Conus striatus* and *Conus catus* (Kohn et al., 1999) had a cusp angle of θ≈21.6 and 22.7 deg (average of 2 species), respectively, while *Conus californicus* (Franklin et al., 2007) had a cusp angle of θ≈25.9 deg. These values were averaged to derive an angle of θ≈23.2 deg, which was subsequently rounded to ∼23.0 deg for testing purposes and fell well within the estimated mean±s.d. tip cusp angle across eight species of cone snails (Kohn et al., 1999): *r*≈24*.*5±6*.*6 deg (Zhang and Anderson, 2023). The biologically relevant dynamic puncture rates were 18*.*5±1*.*1 m s^−1^ (mean±s.d.) for constrained layered salmon tissues and 3*.*7±0*.*4, 5*.*5±0*.*4, 10*.*9±0*.*3, 15*.*8±0*.*7 and 21*.*1±0*.*2 m s^−1^ (means±s.d. of five selected rates) for unconstrained tilapia samples. They were controlled by the pressure and the projectile's loading position within the air cannon and calibrated using high-speed imaging techniques (FASTCAM SA-Z, Photron Inc., 2000–20,000 frames s^−1^) across all samples tested.

Dynamic puncture tests were performed on unconstrained tilapia samples using the experimental setup illustrated in Fig. 1. The samples were suspended using a fishing line attached to two pivot points along the dorsal and ventral sides of the specimen. This configuration was carefully positioned to minimize irregularities in motion and allow for controlled pendulum-like movements upon impact.

### Characterization of fracture surface

The images of punctured samples produced during dynamic puncture tests (e.g. Fig. 1E,F) were captured using a stereo microscope (M205C, Leica Microsystems Inc.). The fracture surface was dyed with blue food coloring before being exposed by cross-sectioning to increase visibility and contrast with the surrounding tissues. The vertex of the fracture surface in Fig. 1E,F was determined by identifying and extrapolating the edge lines (black dashed lines). The maximum depth of puncture (*D*_max_) represents the distance between the superficial opening and the extrapolated apex of the puncture surface determined along its central axis. The mean and standard deviation of *D*_max_ were calculated based on measurements collected by post-processing using ImageJ (Zhang and Anderson, 2022, 2023).

## RESULTS AND DISCUSSION

Cone snails are effective high-speed predators that actively hunt fish. To accomplish this, their puncture systems must overcome several barriers: (1) fish skin acts as a defense against puncture (Zhu et al., 2013; Rawat et al., 2021; Dastjerdi and Barthelat, 2015; Bruet et al., 2008; Allison et al., 2013; Zolotovsky et al., 2021; Minicozzi et al., 2019) and (2) unconstrained fish targets in water may reduce the energy available for penetration through momentum transfer (Anderson et al., 2016, 2019; Zhang and Anderson, 2022). In this section, we quantify the ability of fish skin to mitigate damage from high-speed puncture, as well as the effect of an unconstrained target on overall puncture performance.

### Dynamic puncture resistance of fish skins

Representative images in Fig. 1E,F illustrate the extent of damage and fracture morphologies in salmon tissues. In tissues with skin and scales removed, the damage created by the conical puncture tool was significantly more extensive, spreading both deeper along the puncture direction and wider perpendicular to it, compared with unprocessed tissues. This corresponds to an increased damage reduction capability in the presence of a layered integumentary structure (Zhang et al., 2024). Fig. 2 presents a box and whisker plot comparing the *D*_max_ for the two different salmon samples: with and without the skin layer. Statistical analysis using a two-sample *t*-test revealed significant differences in *D*_max_ between the two conditions (*P<*0*.*0001), with an *F-*value of 6.39. The drastic difference in the average *D*_max_ values (25*.*2±2*.*6 mm for the skinless samples versus 6*.*9±1*.*0 mm for samples with skin) indicates a relative magnitude of damage reduction by ∼72.6%. The standard deviation value for the salmon samples suggests greater variability in *D*_max_ when the outermost layer is removed (s.d.=2.6 mm), compared with samples with skin intact (s.d.=1.0 mm). This change in variability could be due to the inherent heterogeneity and variations in subcutaneous tissue between samples (Anderson et al., 2016). Overall, the substantial reduction in *D*_max_ in Fig. 2 demonstrates the significant role of an outer layer of skin and scales in providing resistance to dynamic biological puncture and mitigating damage.

To explore how puncture tool geometry interacts with fish skin during dynamic biological puncture, we estimated the characteristic length of cone snail radular teeth and compared it with fish skin thickness. We speculated that in successful hunts, the radular tooth's hook region likely must penetrate at least the combined thickness of the fish's integument to reach the subcutaneous tissue for harpooning and gripping. These layers include the scales, which have a thickness of ∼300 µm (Vernerey et al., 2014), followed by the epidermis at around ∼400 µm (Vernerey et al., 2014), and the dermis, which varies in thickness from 20 to 170 µm with an average thickness of ∼95 µm (Wright and Blanchard, 2024). The total thickness, ∼95 µm, represents the minimum barrier that the puncture tool must breach to reach the subcutaneous tissue effectively. Given the natural prey of fish-hunting cone snails are typically smaller with thinner integuments, our benchmark represents a conservative estimate. Successful puncture at this thickness suggests even greater efficacy in thinner, more representative targets. In fish-hunting cone snails, the hook regions of their radula, measured from the apex to the base of the barbs, are relatively small in size. For example, in *C. catus* the hook length is ∼0*.*691 mm (Kohn et al., 1999), in *C. californicus* it measures ∼0*.*312 mm (Kohn et al., 1999), and in *C. striatus*, it is ∼3*.*534 mm (Franklin et al., 2007). It is worth noting that *C. californicus* exhibits a generalist feeding strategy, consuming prey from multiple phyla, including annelids, crustaceans, mollusks and sometimes fish (Kohn, 1966). Its radula lacks the hallmark specializations of obligate fish-hunting cones, reinforcing its distinction from piscivorous *Conus* species (Stewart and Gilly, 2005). Based on these data, we derived an average hook region length for representative piscivorous cone snails of ∼1*.*51 mm. Thus, as suggested by the *D*_max_ data from Fig. 3, at a natural puncture rate of ∼19 m s^−1^, the radular harpoon can easily penetrate the outermost layer and cause significant damage to the fish. The size of the hook region relative to the much larger puncture wound size suggests that the radular harpoon not only pierces the fish skin but also potentially hooks onto the muscle tissues, allowing for the snail to pull and fixate its prey effectively (Schulz et al., 2019; Olivera et al., 2015). We note that the fish samples used in this study may vary slightly in mechanical properties from natural prey of cone snails. This necessitates further research on a broader range of species, as material properties influence penetration efficacy (Zhang and Hutchens, 2021).

### Puncture kinematics of an unconstrained target

In aquatic predation, prey often has the capacity to evade predators. Our hypothesis proposes that a sufficiently high speed is required to effectively penetrate the fish with minimal displacement. Although the tilapia sample in our study was suspended on string, the setup was unconstrained and retained a degree of freedom in the direction of the puncture (Fig. 1), and the fish sample was free to move after impact. However, high-speed impact can occur locally within such a short time frame (<1 ms) that the other parts of the fish body have little time to respond, either actively or passively, making the fish essentially behave like a stationary target (Zhang and Anderson, 2024). Fig. 3 shows the results for the puncture performance in unconstrained tilapia samples, measured under varying puncture rates. Fig. 3A depicts the relationship between velocity and *D*_max_, showing a positive correlation that higher velocities correspond to deeper depths of puncture. At higher biologically relevant speeds (∼19 m s^−1^), the cone snail's harpoon penetrates the tilapia skin more easily by overcoming the mechanical resistance of the integument and capitalizing on inertial effects and the propagation speed of stress waves in the tilapia's tissues, before the fish is able to effectively react or displace itself away from the attack (see Movie 1). Fig. 3B illustrates how higher kinetic energy, associated with higher velocities, results in deeper puncture wounds. At an average velocity of 21.1 m s^−1^, the applied kinetic energy was approximately 1 J, and *D*_max_=15*.*96±1*.*39 mm. A substantial amount of energy is required to achieve a deeper and effective puncture as the kinetic energy scales with velocity squared. In contrast, at an average speed of 3.73 m s^−1^, the kinetic energy was only 0.032 J, and *D*_max_=1*.*16±0*.*25 mm. This *D*_max_ magnitude lies in between the thickness of the fish integument (∼0*.*795 mm) and the average characteristic length of the hook region of cone snail harpoons (1.51 mm). Given this depth of puncture, successful punctures at velocities lower than 3.7 m s^−1^ would be unlikely, as the damage would not be sufficient to penetrate the skin layer. Moreover, at lower velocities, the fish is also more likely to mitigate the impact by passively converting the kinetic energy directed toward it into its own movement through momentum transfer (e.g. Movie 2) or by actively responding through the C-start escape reflex, which occurs within a time scale (∼5–10 ms) comparable to that of lower speed puncture (Wakeling, 2001). However, at higher speeds, this damage mitigation becomes ineffective because of the rapid strike within a short time frame. Tissues distant from the impact do not have time to deform, and the fish cannot react or move away, leading to a deeper and more effective puncture.

### Concluding remarks

Predation in aquatic environments involves unique challenges. At lower speeds, the combined effects of momentum transfer and target mobility limit the effectiveness of predatory strike and puncture (Vogel, 2013; Anderson, 2018). However, at sufficiently high speeds, the inertial effect dominates, allowing for a successful puncture similar to the dynamic puncture of a stationary target (Zhang and Anderson, 2022, 2024; Anderson, 2018; Atkins, 2009). Evolutionarily, certain aquatic predators such as cone snails have likely developed strategies to strike at rapid speeds to overcome these challenges (Nüchter et al., 2006; Patek et al., 2004; Anderson, 2018). Our findings show that while fish skin plays a crucial role in reducing puncture damage, the high-speed strategy utilized by cone snails effectively overcomes this mechanical resistance, making them predators capable of immobilizing and harpooning prey with remarkable efficiency.

One limitation of this study is the effect of viscous force of water on puncture performance, which can cause additional energy dissipation during the tool's motion (Anderson, 2018). Further studies should incorporate hydrodynamics to provide a more accurate quantification of the aquatic dynamic puncture behavior. Moreover, our results should be interpreted in light of scaling effects. Compared with the ∼1 mm length scale of cone snail radular teeth, our artificial conical puncture tool is scaled up by roughly a factor of 10, resulting in a lower sharpness when measured by tip radius of curvature. However, the cusp angle – a previously shown better predictor of sharpness (Crofts et al., 2019) – is scale independent. Scaling is a complex issue when dealing with small biological structures. Here, we used prescribed tool geometries to control and isolate scaling effects, allowing insights into the kinematics of biological puncture.

## Supplementary Material

10.1242/jexbio.250634_sup1 Supplementary information
