# Supplementary material for: The biomechanics of fish skin: assessing puncture resistance to the dynamic predatory mechanism of cone snails
Source: J Exp Biol. 2026 Jan 2;229(1):jeb250634. doi: 10.1242/jeb.250634 (PMC12813667; doi:10.1242/jeb.250634)
Supplement: Supplementary information [file jexbio-229-250634-s1.pdf]

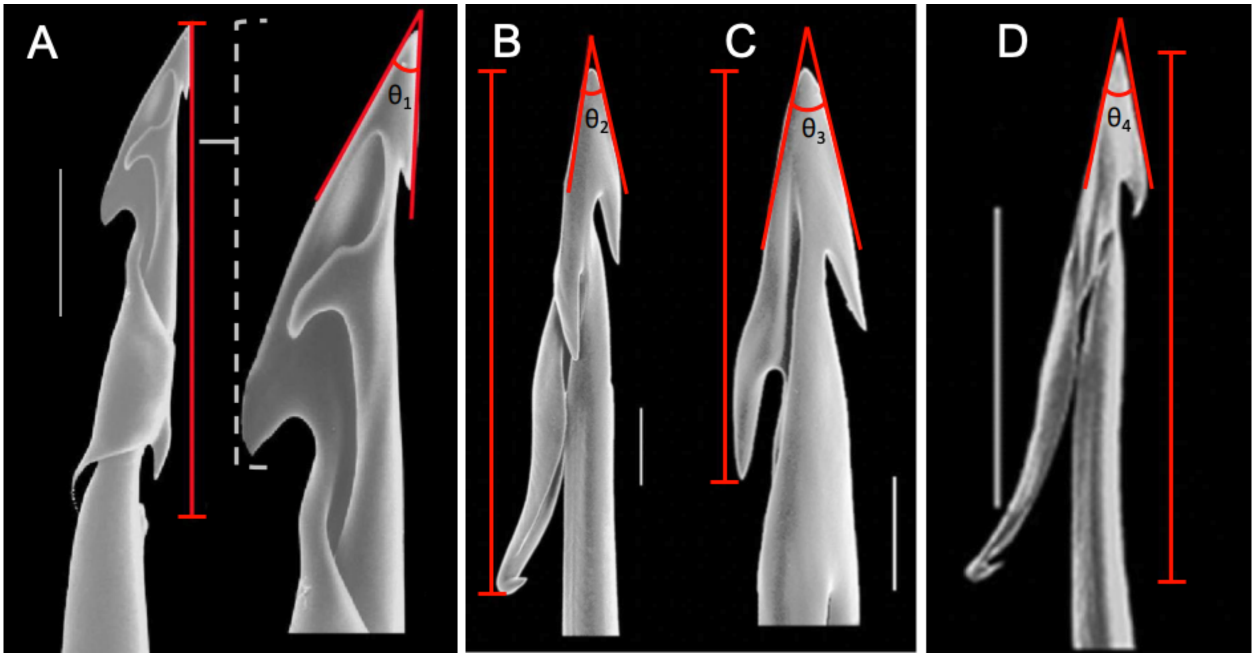

Fig. S1. Measurements of cusp angle and hook region length of radular harpoons from three species of cone snails (from left to right): *C. californicus* (A), *C. catus* (B and C), and *C. striatus* (D). The cusp angle measurement for each species was extracted from SEM images of cone snail radular harpoons [1, 2] using ImageJ. Scale bars: A = 0.1 mm; B = 0.1 mm; C = 0.1 mm; D = 2 mm. Average cusp angles: $\theta_1 = 25.9°$, $\theta_2 = 20.7°$, $\theta_3 = 24.6°$, $\theta_4 = 21.6°$. The length of the hook region is determined from the apex to the base of the barbs. Measurements of hook region lengths: *C. catus*: ≈ 0.691 mm; *C. californicus*: ≈ 0.312 mm; and *C. striatus*: ≈ 3.534 mm. Mean and SD: 1.51 ± 1.44 mm.

Journal of Experimental Biology • Supplementary information

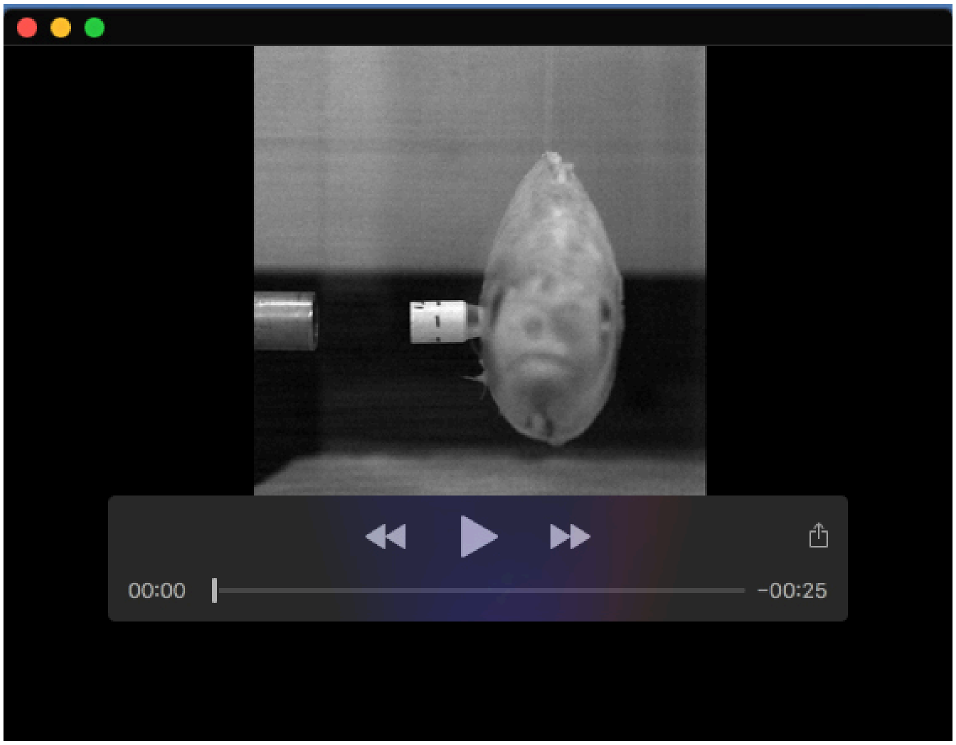

Movie 1. Dynamic puncture experiment on unconstrained fish target at a higher puncture rate. Average speed at impact: 21.1 m/s. Frame rate of high-speed imaging: 20000 fps.

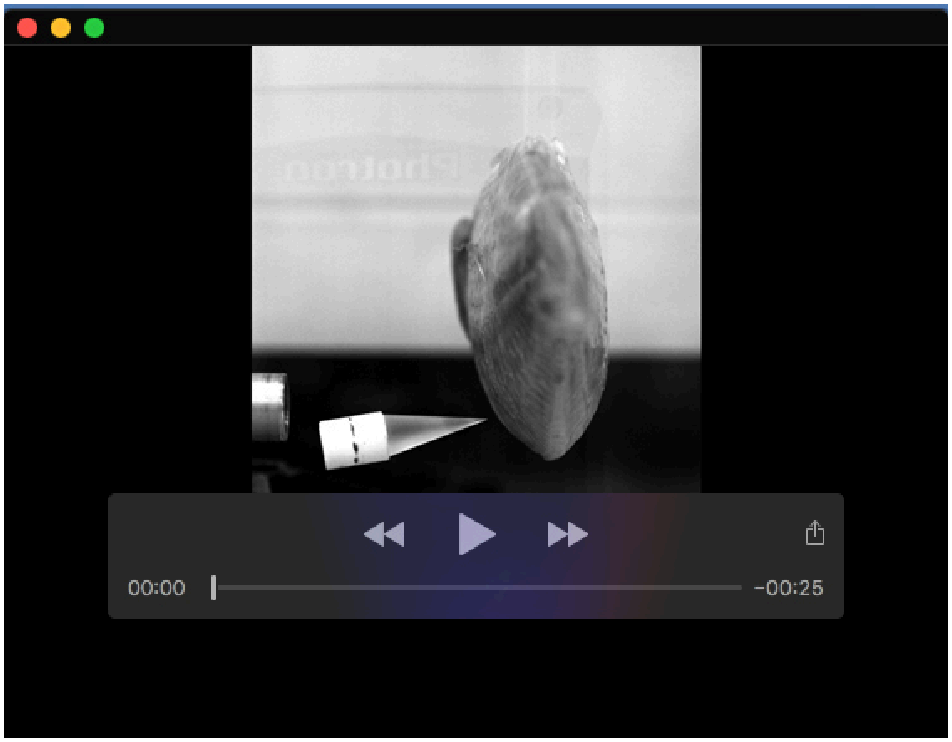

Movie 2. Dynamic puncture experiment on unconstrained fish target at a lower puncture rate. Average speed at impact: 3.73 m/s. Frame rate of high-speed imaging: 2000 fps.

Journal of Experimental Biology • Supplementary information

References

[1] Kohn A. J., Nishi M., Pernet B. 1999. Snail spears and scimitars: a character analysis of *Conus* radular teeth. *Journal of Molluscan Studies* **65**, 461–481. (10.1093/mollus/65.4.461)

[2] Franklin J. B., Fernando S. A., Chalke B., Krishnan K. 2007. Radular morphology of *Conus* (Gastropoda: Caenogastropoda: Conidae) from India. *Molluscan Research* **27**, 111–122. (10.11646/mr.27.3.1)
